# Impact of Water Adsorption on Nonlinear Optical Properties of Functionalized Porous Silicon

**DOI:** 10.1186/s11671-016-1805-y

**Published:** 2017-01-23

**Authors:** Andrii V. Uklein, Volodymyr V. Multian, Bogdan V. Oliinyk, Volodymyr V. Doroshchuk, Sergei A. Alekseev, Volodymyr V. Lysenko, Mykhailo S. Brodyn, Volodymyr Ya. Gayvoronsky

**Affiliations:** 1 Institute of Physics NAS of Ukraine, Prospect Nauky 46, Kyiv, 03028 Ukraine; 2 Taras Shevchenko National University of Kyiv, 64 Volodymyrska Str., Kyiv, 01601 Ukraine; 3 Lyon Institute of Nanotechnologies, INL, CNRS UMR-5270, INSA de Lyon, 7 avenue Jean Capelle, Bat. Blaise Pascal, Lyon, 69621 Villeurbanne cedex France

**Keywords:** Porous silicon functionalization, Nonlinear optical response, Water adsorption/desorption, Surface diagnostics

## Abstract

The porous silicon (PS) surface modification diagnostics due to functionalization and water adsorption/desorption processes were provided by the self-action effects of picosecond range pulsed laser radiation at 1064 nm. It was shown that the PS surface functionalization—oxide removal, alkylation, and oxidation—resulted in a refractive nonlinear optical (NLO) response sign turn to self-focusing (*Δn*>0) versus the self-defocusing (*Δn*<0) observed in the aged PS. The sensitivity of the proposed technique was revealed to water adsorption/desorption from the chemically oxidized PS interface. For the dried PS, the self-defocusing effect with corresponding NLO cubic susceptibility Re(*χ* ^(3)^)∼−4.7·10^−9^ esu was observed versus the self-focusing one (∼5·10^−8^ esu) for the PS positioned in saturated water vapor at room temperature. The obtained results demonstrate high sensitivity and wide versatility of the proposed readout technique based on pulsed laser radiation self-action at 1064 nm to the PS surface modification monitoring/diagnostics applications.

## Background

Porous silicon is a well-known material for applications in microelectronics, optoelectronics, energy conversion, etc. [1]. An interesting example is the field of Si sensors. Multiple applications of the PS in sensors technology are mainly based on significant electrical or optical properties changes [2, 3] due to the adsorption of the molecules for gas, humidity, biological sensing, and others [4].

The mean objective of the sensor is to produce the discrete/continuous signal that is sensitive to the presence of the corresponding analyte. The particular properties of the PS can be efficiently used to convert the effects of analytes into electrical or optical signals. Hence, the stabilization of PS via a suitable surface treatment is an indispensable step for fabricating a stable sensor device. The oxidation is among the techniques that are commonly used to functionalize PS surface. However, the porous SiO_2_ structure remains prone to degradation in aqueous media. Higher stability of functionalized surface was achieved by the formation of silicon carbon bonds and consecutive blocking of water access to the surface as well as hampering of surface bonds nucleophilic attack by water or hydroxide. Such functionalization of the PS significantly impacts the electronic surface states, particularly saturates the dangling bonds that are normally located on PS surface (1 bond per 10^4^−10^5^ atoms), and consequently changes the PS properties [5]. In recent studies of mesoporous Si [6], a high sensitivity of the laser pulses self-action effects to the surface electronic states excitation that related to the defects in the thin silicon oxide layer at the boundaries of nanocrystals and pores was shown. According to those results, in this paper we propose a new method based on monitoring of PS NLO response for sensing application. The photoinduced variation of the refractive index demonstrated a high sensitivity to the PS surface modifications such as functionalization and to water adsorption/desorption.

## Methods

### Porous Silicon Fabrication, Oxidation, and Chemical Functionalization

Porous silicon was prepared by anodic etching of p ^++^ type boron-doped (0.001–0.003 *Ω*·cm) double-side polished (100)-oriented silicon wafers. The anodization was performed in a Teflon®; cell with an Au counter electrode and backside Cu electrode. Silicon samples were etched for 10 min within 150 mA ·cm^−2^ anodic current density in a solution containing 1:1 volume mixture of HF (49%) and ethanol. A permanent stirring of the etching solution was used to evacuate hydrogen bubbles formed during the anodization process. Two-second etch stop intervals (2 s etch + 2 s stop) were introduced during the anodization in order to replenish the electrolyte in depth of the nano-pores and thus to avoid a porosity gradient along the layer thickness. At the end of the anodization process, formed PS layers were removed from the bulk silicon wafer by switching anodization current to electropolishing regime (0.5 A ·cm^−2^ current density pulse during 5 s) to form so-called free-standing PS layer, further referenced as PS sample. Values of thickness and gravimetric porosity for all PS layers used in this work were found to be 37 ±1 *μ*m and 64 ±2%, respectively.

The surface of the PS after storage in ambient air underwent partial oxidation. To remove the surface oxide, the PS samples were dipped in HF:EtOH (1:9) for 10 min, afterwards rinsed with anhydrous ethanol and hexane and dried in an Ar stream. Resulted oxide-free PS (refreshed PS, ref-PS) was used for measurements or further chemical modifications immediately after the preparation.

**Fig. 1:**
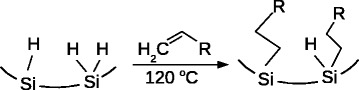
Schematic representation of the organic fragments grafting onto the PS internal surface

Grafting of the organic fragments onto the PS internal surface was achieved via thermal hydrosilylation approach (Fig. 1). The ref-PS was treated with neat 1-decene or 1-octadecene at 120 °C for 12 h under the Ar atmosphere to get PS-C10 and PS-C18 samples.

After the reactions, the samples were rinsed with hexane and dried in an Ar stream.

The oxidation of the PS to get PS-ox sample was performed by dipping of the PS into as-prepared piranha mixture (H_2_O_2_(30%):H_2_SO_4_ 3:7 *v*:*v*) for 15 min that was followed by water and ethanol rinsing and drying in ambient air.

### Characterization Methods

The transmittance spectra of the PS samples were recorded in three ranges: (i) 400–7000 cm ^−1^—Nicolet Nexus 470 spectrometer; (ii) 8000–12,000 cm ^−1^—MDR-6 LOMO monochromator; (iii) 11,000–20,000 cm ^−1^—Evolution 600 Thermo Scientific spectrometer.

All spectra were recorded at room temperature in ambient conditions. In order to be able to compare intensities of the spectral peaks, the area of the open space used for the detection of background transmitted signals was always kept the same as the area of the PS samples exposed to the laser radiation. Interpretation of spectral data and calculation of volume fractions of grafted groups, SiO_2_ or water in the pores were performed according to [7].

The optical scattering indicatrices into forward and backward hemispheres of the PS samples were measured under the CW DPSS laser irradiation at 1064 nm. The scattered light was registered by the CCD camera (ATiK 16 IC-HS with pixel size 7.4×7.4 *μ*m^2^ and 640×480 image resolution) at the distance of 39.2 cm. From the obtained results, the scattering losses into forward and backward hemispheres were estimated within the approach described in [8].

The NLO response due to the self-action of the picosecond range laser pulses at 1064 nm (42 ps FWHM, repetition rate 50 Hz) in the samples was studied within the laser beam spatial profile analysis in the far field [9]. The proposed method was successfully applied for the porous silicon NLO response studies [6]. In this paper the obtained total and on-axis transmittance dependencies are presented as the smoothed curves produced by local B-splines of measured data. Each curve corresponds to the ∼5000 registered laser shots. The detailed models for the cubic NLO susceptibility (*χ* ^(3)^) calculation from the experimental data are given in [9]. The real part characterizes the photoinduced variations of the refractive index *Δn*∼Re(*χ* ^(3)^)*I* within the peak laser intensity *I*.

It should be noted that all optical and NLO experiments were performed through the fixed diaphragm on the samples in order to analyze the signals from the same area.

## Results and Discussion

### Optical Characterization

#### Spectral Analysis

**Fig. 2:**
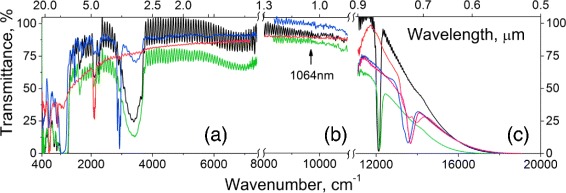
Transmittance FTIR (**a**), NIR (**b**), and UV-vis (**c**) spectra of functionalized porous silicon: PS (*black curve*), PS-ox (*green*), ref-PS (*red*), PS-C10 (*pink*), and PS-C18 (*blue*)

Typical transmittance FTIR spectra of the studied functionalized PS free layers are shown in Fig. 2 a. The sinusoidal fringes related to the interference of the infra-red light on homogeneously thin PS layers can be clearly seen at all spectra. The values of the PS refractive index, derived from these fringes in a 4000–6000 cm ^−1^ range, were used for calculation of porosity and volume fraction of chemical groups as it was described in [7]; corresponding data are presented in Table 1. The results coincide to the magnitudes calculated by the same approach from the (ii) spectral range presented in Fig. 2 b.

**Table 1:** Surface groups and quantitative composition for the samples of the functionalized porous silicon

No	Treatment	Sample	Surface layer	*f*_Si_, %	*f*_R_, %	*f*_por_, %
1	*Aged*	PS	SiH _x_, O_3_SiH, SiOH	31.5	–	68.5
1’	*Refreshed*	PS-ref	SiH _x_	22.6	–	77.4
2	*Alkylation*	PS-C10	-(CH_2_)_9_CH_3_	22.2	9.7	68.1
3		PS-C18	-(CH_2_)_17_CH_3_	22.6	18.4	59.0
4	*Chem. oxidized*	PS-ox	SiO_2_ (SiOH)	28.8	6.1	65.1
		PS-ox-H_2_O	SiO_2_ (SiOH)	28.8	6.1	65.1 ^a^

^a^Pore fraction 65.1% consists 48.2% of H_2_O and 16.9% of voids

The *f*_Si_, *f*_R_, and *f*_por_ are the volume fractions of the Si, functional groups and empty pores correspondingly

As it can be seen from the spectral bands of the PS sample, its storage in ambient conditions for continuous (few month) time resulted in a significant oxidation of the surface. Besides the silane groups (Si _4−x_ *SiH*_x_, *x* = 1, 2 or 3; *ν*(SiH_3_) at 2137 cm ^−1^, *ν*(SiH_2_) at 2114 cm ^−1^, *ν*(SiH) at 2088 cm ^−1^, *δ*(SiH_2_) at 906 cm ^−1^, *ω*(SiH_2_, at 660 cm ^−1^ and *ω*(SiH) at 625 cm ^−1^ according to [10, 11]), significant fraction of oxidized species, particularly the Si–O bonds (*ν*(Si-O) at 1200–1000 cm ^−1^), O_3_SiH fragments (*ν*(SiH) at 2264 cm ^−1^, and *ω*(SiH) at 875 cm ^−1^) and silanol groups, which physisorb water molecules (wide *ν*(O–H) band at 3400 cm ^−1^), are present. This fact gives reasonable explanation of the mismatch between the values of the PS porosity, found by gravimetric (63%) approach and refractometry technique via interferometric pattern analysis (68.5%) with accounts of only two component effective media—silicon and voids. The oxidation of the PS resulted in overall reduction of the refractive index and increase of “refractometric” porosity value. More precise and reliable multi-component analysis of the PS functionalization [12] should include dispersion of the polarizabilities of the grafted layer.

**Fig. 3:**
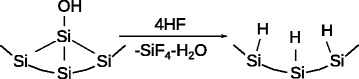
Schematic representation of oxidized surface species removal from Si surface under the treatment with HF

Treatment with the HF resulted in almost complete removal of oxidized surface species (Fig. 3); only the silane groups’ bands could be seen in the spectrum.

The spectral baseline of the oxide-free ref-PS drops down with decrease of the wavenumber. Probably this takes place due to the release of charge carriers (holes), trapped onto the surface defects in initial and functionalized PS samples. Significant increase of the porosity under the HF treatment indicates that “refresh” procedure resulted not only in oxide removal but also in dissolution of the silicon, probably, of the defect-enriched subsurface layers. A similar phenomenon, i.e., Si surface defects removal under the action of HF accompanied with the conductivity increase, is described in our recent work [13].

Covalent grafting of alkyl groups in the PS-C10 and PS-C18 samples via hydrosilylation reaction (Fig. 1) is confirmed by the presence of the characteristic bands: *ν*_as_ (CH_3_) at 2962 cm ^−1^, *ν*_as_ (CH_2_) at 2925 cm ^−1^, *ν*_s_ (CH_2_) at 2853 cm ^−1^, *δ*(CH_2_) at 1467 cm ^−1^, and *ρ*(CH_2_) at 720 cm ^−1^. Absence of sharp *ν*(C=C) band at 1645 cm ^−1^ in the spectra of all studied samples proved an absence of physisorbed alkenes, i.e., all organic groups are grafted covalently according to the scheme in Fig. 1. Significant reduction of the SiH _x_ bands intensities at the spectra of PS samples with grafted organic groups compared to the spectrum of the ref-PS indicates partial consumption of the SiH _x_ groups in the reaction course. The volume fraction of grafted alkyl groups (Table 1) corresponds to their concentration in a range of 0.7–0.8 mmol/cm^3^, correlating well with the values, preciously found in [7]. Despite the hydrosilylation proceeding in an Ar atmosphere, it is accompanied by partial oxidation of the PS surface, as the bands of oxidized Si species are present in the spectra of all hydrosilylated samples. The oxidation is caused by the presence of the traces of O_2_ and H_2_O in the reaction mixture; it should result in a restoration of the surface defects and it should not be neglected in the consideration of the PS NLO properties.

The chemical oxidation of the PS with piranha resulted in almost complete oxidation of Si _4−x_SiH_*x*_ and O_3_SiH groups and formation of hydroxylated SiO_2_ surface layer with corresponding bands manifestation (silanol groups: *ν*(O–H) isolated at 3745 cm ^−1^ ; SiO_4_ units: *ν* as (O–Si–O) at 1200–900 cm ^−1^, *ν*_s_(O–Si–O) at 810 cm ^−1^, and *δ*(O–Si–O) at 470 cm ^−1^) which strongly absorbs water (broad *ν*(O–H) band at 3400 cm ^−1^, and *δ*(H–O–H) at 1640 cm ^−1^).

Continuous treatment of the PS-ox in saturated water vapors results in pores filling with H_2_O due to the capillary condensation. However, we have found a coincidence of the refractive indexes of the PS-ox left in the ambient conditions and the dried sample at 130 °C in an Ar flow. It means that at small partial pressure the H_2_O mainly forms adsorption complexes onto the Si–OH groups, while liquid condensation in the pores starts at higher vapor pressure.

In the UV-vis range (Fig. 2 c), the spectra of studied samples can be presented as the constantly descending curves with the wavelength decrease (Fig. 2 c) that is typical for the thin layers of silicon. The optical bandgap *E*_g_ corresponds to 1.19 ±0.02 eV, being estimated by linear approximation of (*α*(*hν*)*hν*)^1/2^ versus energy quanta *hν* dependence in a 1.45–2-eV range, where *α* is the optical absorption coefficient. No impact of the chemical functionalization on *E*_g_ was found. For the studied samples with average sizes of Si nanocrystals/pores ∼10/20 nm correspondingly, the obtained *E*_g_ exceeds the bandgap for a bulk Si (*E*_g_=1.12 eV), probably, due to the pronounced manifestation of the direct transitions within porosity growth and a quantum confinement effect in Si nanocrystallites with sizes less than 10 nm that form the porous silicon. The obtained optical bandgap is overlapping with the filled with electron deep trap position of 1.2 ±0.09 eV at the SiO_2_-bulk Si interface [14] that can play an important role due to the developed surface of the studied PS samples.

Relatively sharp dip found in all the spectra in a 700–900-nm range has a purely optical origin. It appears due to the interference of light in a sub-micron highly porous film, formed on the outer surface of the PS free layer during the stage of its detachment by a high current pulse. As the spectral position of the observed interference dips are far beyond the laser wavelength 1064 nm used for the NLO property characterizations, the mentioned thin layer impact on optical response can be omitted for transmission geometry readout.

#### Elastic Optical Scattering Analysis

**Fig. 4:**
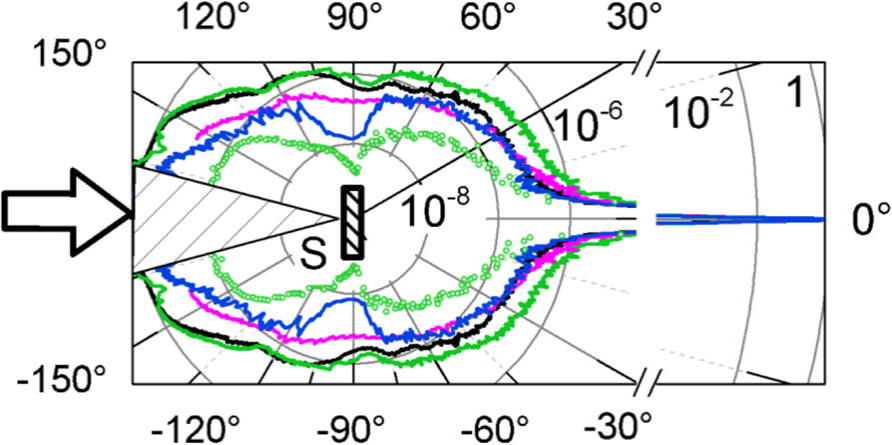
Indicatrices of the elastic light scattering in forward and backward hemispheres for functionalized porous silicon samples at 1064 nm: PS (*black curve*), PS-C10 (*pink*), PS-C18 (*blue*), and PS-ox (*green*). The *green dots* present the elastic light scattering of PS-ox sample in saturated H_2_O vapor

The elastic scattering indicatrixes at 1064 nm were measured in order to estimate the effect of the PS surface functionalization on optical quality and dispersivity of the samples. The polar plots of the cross sections of the indicatrices are presented in Fig. 4. It was shown that the alkylation of the PS surface does not deteriorate the dispersivity of the PS in the scattering angle range |*θ*|≤30° and even suppressed it for the larger angles at about factor 1.5–2 and more than order of magnitude in a backward hemisphere (|*θ*|>90°). The sample PS-C18 with longer alkyl chain (blue curve) has reduced dispersivity and more pronounced interference pattern at large |*θ*| versus the PS-C10 (pink one) where detected signal level is less than six orders of the input beam magnitude. For the small angles |*θ*|≤3°, the scattering indicatrix of the PS-ox (green) is similar to the aged-PS one, while its dispersivity rises at about factor 2.5 for the rest forward hemisphere. In backward hemisphere, the angular dependencies of the scattered signal are similar for the aged-PS and PS-ox except narrow dips for the PS-ox signal at |*θ*|∼160°. The indicatrix of the ref-PS is close to the PS-ox and is not presented in Fig. 4.

We have processed experimental data and estimated the scattering losses into forward and backward hemispheres. The results are presented in Table 2. One can see that the scattering losses of the aged-PS, PS-10, and PS-C18 in the forward hemisphere are almost equal within the experimental error (±0.05%) and about 75% higher versus the PS-ox. The obtained estimates reflect the fact that the evident differences of the scattering indicatrix in the large scattering angles range does not effect on the integrated metrics due to the drastic difference in signal level (more than six orders less) in comparison with small-angle scattering impact for the low dispersive samples.

**Table 2:** The real part of the cubic NLO susceptibility Re(*χ* ^(3)^), the peak laser intensity magnitude of the first extremum *I*_ex_, and scattering losses into forward (FH) and backward (BH) hemispheres at 1064 nm for the samples of functionalized porous silicon

No.	Sample	*I*_ex_, MW/cm ^2^	$\text {Im}(\chi ^{(3)}_{\text {eff}}), \times 10^{-11}$ esu	Re(*χ* ^(3)^), ×10^−8^ esu		Scattering losses, %	
			25–50 MW/cm^2^	25–50 MW/cm^2^	190–240 MW/cm2	FH	BH
1	Aged-PS	82.6	−1.7	−1.0	−0.5	0.4	0.5
1’	Ref-PS	50.7	1.1	2.0	−0.2	0.6	0.4
2	PS-C10	42.3	−0.2	0.2	−0.1	0.4	0.2
3	PS-C18	115.1	5.3	1.9	−0.2	0.4	0.1
4	PS-ox	167.2	−1.2	0.6	−0.2	0.7	0.6

In the backward hemisphere, the alkylation results in up to 2.5 times reduction of the scattering losses for the PS-C10 and up to 5 for the PS-C18.

Either, the impact of the water adsorption on the elastic light scattering of the oxidized PS was studied. The corresponding scattering indicatrix for the PS-ox sample in saturated H_2_O vapor is presented in Fig. 4 as green dots. It is shown that the pore filling with water results in significant reduction of the light scattering in both forward and backward hemispheres. The scattering losses are 3.5 times less in the forward and 6 times less in the backward hemisphere versus the PS-ox ones. The obtained results indicate that the elastic scattering analysis in near IR range can be applied as a sensitive indicator of the PS surface functionalization and water adsorption effects on it due to the high sensitivity in more than seven orders of magnitude signal range.

### Nonlinear Optical Response

**Fig. 5:**
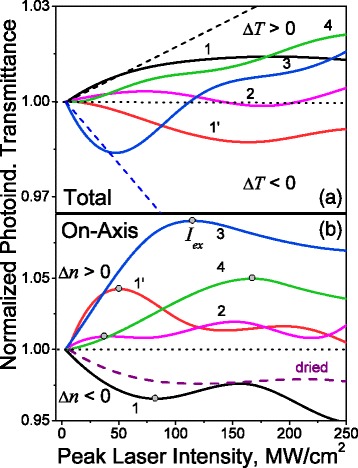
The photoinduced variations of the normalized total (**a**) and on-axis (**b**) transmittances versus the peak laser intensity of picosecond laser pulses at 1064 nm for the samples of functionalized porous silicon: 1 – PS (*black curve*), 1’ – ref-PS (*red*), 2 – PS-C10 (*pink*), 3 – PS-C18 (*blue*), and 4 – PS-ox (*green*). The *dashed lines* in **a** represent the results of the theoretical modeling for the samples PS and PS-C10 by the formulae (1). The *dashed curve* in **b** corresponds to the oxidized sample after drying exposure of 120 h

The photoinduced variations of the normalized total and on-axis transmittances for the studied samples are presented in Fig. 5 a, b correspondingly. The relative error of the curves is about ±0.2% for the total transmittance dependencies and ±1% for the on-axis ones.

It is shown that the initial PS demonstrates the photoinduced bleaching effect that saturates at about 175 MW/cm^2^. The surface functionalization results in absorptive NLO response sign turn after the refreshing (curve 1’) or longer alkyl chain grafting (curve 3). The almost total compensation of the PS NLO response was observed for the sample PS-C10 with shorter alkyl chain (curve 2). The oxidized sample demonstrates the monotonic photobleaching effect. The calculated effective $\text {Im}(\chi ^{(3)}_{\text {eff}})$ magnitudes due to the approach described in [5] are presented in Table 2.

The on-axis transmittance dependencies of the samples are not monotonic (see Fig. 5 b) and can be characterized by the peak intensity magnitude of the local extrema *I*_ex_. The *I*_ex_ of the first extrema are presented in Table 2. It is shown that the aged-PS sample (curve 1) demonstrates the self-defocusing effect in the peak laser intensity range *I*<*I*_ex_ that corresponds to the negative photoinduced variations of the refractive index *Δn*<0. At higher peak intensities, the effect saturates and turns to self-focusing. The surface functionalization (refreshing/oxidation/alkylation) results in refractive NLO response sign turn to the opposite: to the self-focusing effect in the range *I*<*I*_ex_ and to the self-defocusing one for the range *I*>*I*_ex_.

From the obtained dependencies, the real part of the cubic NLO susceptibility was calculated for two peak laser intensity ranges: (i) 25–50 MW/cm^2^ and (ii) 190–240 MW/cm^2^. The results are presented in Table 2. It can be seen that in the first range the aged-PS demonstrates the negative Re(*χ* ^(3)^)∼−1.0×10^−8^ esu versus the functionalized samples with Re(*χ* ^(3)^)>0. The PS surface refreshing results in the refractive NLO response efficiency enhancement up to two times |Re(*χ* ^(3)^)_ref-PS_|∼2|Re(*χ* ^(3)^)_PS_|, whereas the oxidation–in reduction |Re(*χ* ^(3)^)_PS-ox_|∼0.6|Re(*χ* ^(3)^)_PS_|. The alkyl chain grafting on the PS surface leads to the almost total compensation of its refractive NLO response for shorter chain length (PS-C10). For the sample with longer alkyl chain (PS-C18), the NLO response efficiency enhancement similar to the ref-PS was observed.

For all samples the refractive NLO response over the absorptive one |Re(*χ* ^(3)^)/Im(*χ* ^(3)^)|∼10^3^−10^4^ that is promising for the photonics applications.

Let’s study the impact of the pronounced photoinduced refractive index variations on total transmittance of the samples. In this case, the sample is considered as nonlinear Fabry-Perot interferometer [15]. The photoinduced total transmittance variations of the interferometer can be presented as the expansion into *φ*(*I*):

1 $$ T(I) = {K^{T}_{0}} + \frac{1}{\sqrt{2}}\varphi (I) {K^{T}_{1}} + \frac{1}{2}\varphi^{2}(I) {K^{T}_{2}}+\dots,  $$

where *φ*(*I*) is the photoinduced phase shift after the sample, ${K^{T}_{m}}$ are coefficients that are determined by the interferometer parameters (thickness, refractive index, reflection) [16]:

2 $$ \begin{aligned} & {K^{T}_{0}} = \frac{T_{lin}}{1+\rho^{2}-2\rho \text{cos}(\phi)},\\ & {K^{T}_{1}} = \frac{2({K^{T}_{0}})^{2} \rho \text{sin}(\phi)}{T_{lin}},\\ & {K^{T}_{2}} = \frac{({K^{T}_{0}})^{2}}{3T_{lin}}\left[(1+\rho^{2}-2\rho\text{cos}(\phi))-{\vphantom{\frac{2^{a^{a}}}{2}}}\right.\\ &\left.\qquad-\frac{{K^{T}_{0}}(1+2\rho^{2} (\text{cos}(2\phi) -6) + \rho^{4} + 4 \rho(1+\rho^{2})\text{cos}(\phi))}{T_{lin}} \right], \end{aligned}  $$

where $\rho =\sqrt {R_{12}R_{23}}$, *ϕ*=*kn*_0_ *L*, *R*_12_ and *R*_23_ are the reflection coefficients of the input and output mirrors of the interferometer, *T*_*lin*_ is the transmittance coefficient without interference impact, *k* is the wavenumber, *n*_0_ is the refractive index, and *L* is the thickness of the interferometer.

The results of the theoretical modeling for the samples PS and PS-C10 by the formulae (1) are presented in the Fig. 5 a with dashed lines. One can see that for the initial peak laser intensity range <50 MW/cm^2^ the measured photoinduced total transmittances coincide to the ones obtained for the nonlinear Fabry-Perot interferometer with corresponding photoinduced phase shift. The similar calculations for the other samples also confirmed that the observed photoinduced variations of the total transmittance in the studied functionalized PS are mostly determined by the photoinduced variations of the refractive index.

In order to study the influence of adsorbed water on the NLO properties of the PS layers, the PS-ox sample in the holder was placed inside a hermetically closed 5-mm glass cuvette above the layer of anhydrous CaCl_2_, which is an efficient water absorber, and afterwards above the layer of H_2_O.

**Fig. 6:**
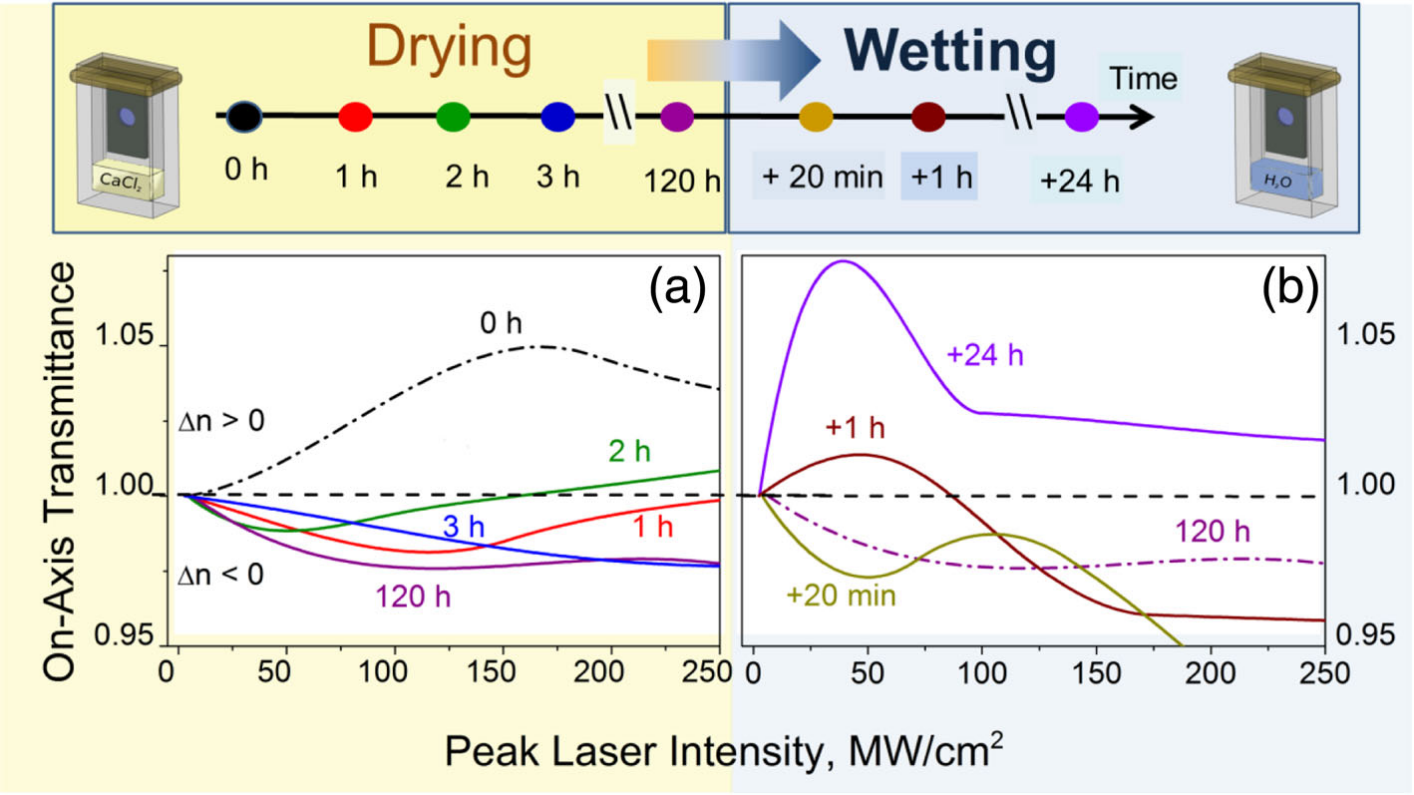
The photoinduced variations of the on-axis transmittances versus the peak laser intensity at 1064 nm for the oxidized porous silicon PS-ox in cuvette with: **a** CaCl_2_ (drying); **b** saturated H_2_O vapor (wetting). The sample exposition for drying was: 0 h *black curve*, 1 h *red*, 2 h *green*, 3 h *blue*, 120 h – *purple*; for wetting: 120 h + 20 min *dark yellow curve*, +1 h *wine*, +24 h *violet*

Initially, the NLO response measurements were performed for the sample placed in the empty cuvette (0 h). Next the CaCl_2_ was introduced into the cuvette and it was hermetically closed. The following measurements were done after 1, 2, 3, and 120 h of drying. The corresponding dependencies of the photoinduced on-axis transmittance are presented in Fig. 6 a. It can be seen that the pronounced self-focusing effect for the PS-ox sample under initial conditions (air humidity ∼75%) for peak intensity range *I*<*I*_ex_ turns to self-defocusing one after drying. The efficiency of the process and *I*_ex_ depend on the sample exposition for drying. The corresponding Re(*χ* ^(3)^) and *I*_ex_ magnitudes are presented in Table 3. It is shown that the water desorption from the PS-ox surface during 2 h causes the |Re(*χ* ^(3)^)| rise with reduction of *I*_ex_. After the 3 h, the |Re(*χ* ^(3)^)| reduces with corresponding *I*_ex_ rise. For the dried sample, the Re(*χ* ^(3)^)≈−4.7×10^−9^ esu and *I*_ex_=119.1 MW/cm^2^ was established. For the range *I*>*I*_ex_, the similar tendency for |Re(*χ* ^(3)^)| was observed with saturation of the effects for the dried PS-ox.

**Table 3:** The real part of the cubic NLO susceptibility Re(*χ* ^(3)^), the peak laser intensity magnitude of the first extremum *I*_ex_, and scattering losses for oxidized porous silicon PS-ox in atmosphere with different water concentrations

Cuvette with:	Time, h	*I*_ex_, MW/cm^2^	Re(*χ* ^(3)^), ×10^−9^ esu		Scattering losses, %	
			25–50 MW/cm^2^	190–240 MW/cm^2^	FH	BH
–	0	167.2	6.1	−2.0	0.7	0.6
Drying with CaCl_2_	1	115.9	−3.6	1.0		
	2	50.1	−4.1	1.1		
	3	263.0	−1.8	−0.2		
	120	119.1	−4.7	<0.1		
Saturated H_2_O vapor	+1/3	50.4	−6.3	−7.3		
	+1	45.2	6.1	−0.4		
	+24	39.7	49.7	−0.9	0.2	0.1

In order to study the impact of water adsorption on the refractive NLO response of the PS-ox, the sealed cuvette was unpacked and the powder of CaCl_2_ was removed. We filled the bottom part of the cuvette with water (it did not contact directly the sample) and sealed the cuvette again. The measurements were provided after 20 min, 1 h, and 24 h of the sample exposition in saturated water vapor at room temperature. The water adsorption on the PS-ox surface results in reverse sign turn of the on-axis transmittance from the self-defocusing observed for dried sample to the self-focusing. After the 24-h exposition, about 8 times enhancement of the refractive NLO response (Re(*χ* ^(3)^)∼49.7×10^−9^ esu) versus the initial one before the treatment route was observed. The *I*_ex_ reduction with water adsorption on the PS-ox surface was revealed.

The obtained results indicate high sensitivity of the photoinduced refractive index variations to the water adsorption/desorption on the PS surface.

### Discussion

The PS surface functionalization due to refreshing, alkylation, oxidation, and water adsorption/desorption effects on transformation of the refractive NLO response versus the aged-PS one. In general the mentioned functionalization provides turn from the self-defocusing (*Δn*<0) to the self-focusing (*Δn*>0) of the passed through the porous layer laser beam. The opposite turn from *Δn*>0 to the negative one was observed only for the oxidized PS along drying treatment. The wetting of the dried PS surface restores the self-focusing effect with 8 times rise of the refractive NLO response efficiency.

We suggest the following explanation of the specific NLO response transformation due to the redistribution of the delocalized and localized carriers impact in the framework of the Drude model of optical response that is widely applied for the functional materials characterization [17, 18]:

3 $$  \Delta n = n_{2} I + \frac{2\pi e^{2}}{n_{0}}\left[-\frac{f_{\text{vb}} n_{\text{vb}}}{m^{*}_{h}\omega^{2}}-\frac{f_{\text{cb}} n_{\text{cb}}}{m^{*}_{e}\omega^{2}} +\frac{f_{\text{tr}} n_{\text{tr}}}{m(\omega^{2}_{\text{tr}}-\omega^{2})}\right]  $$

The first term *n*_2_ *I*, where *n*_2_ is the nonlinear refractive index, is related to the instantaneous Kerr effect, being observed in all materials due to the virtual transitions impact with low efficiency yield. The second and the third terms correspond to the self-defocusing Drude-type response of the delocalized carriers—holes and electrons—with corresponding effective masses $m_{h}^{*}$ and $m_{e}^{*}$; *f*_vb_/*f*_cb_ are the oscillator strength, *n*_vb_/*n*_cb_ the are densities, and *n*_0_ is the linear refractive index of the material.

The last term corresponds to the NLO response of the localized carriers at the traps, *f*_tr_ is the oscillator strength of the transition between the fundamental and the first excited/delocalized state with corresponding energy difference $\hbar \omega _{\text {tr}}$. For the deep traps, the response is positive in *ω*<*ω*_tr_ domain [17].

For the aged-PS in the studied excitation range, the photoinduced refractive index variation can be attributed to the Drude-type response of the carriers (both holes and electrons) in highly doped with boron p ^++^ silicon bulk. The covering silicon natural oxide shell is close to stoichiometric SiO_2_ [19] with low concentration of the deep traps and low surface density of the silanol groups. Their efficient NLO response can be observed at lower peak laser intensities (<3 MW/cm^2^) that is out of scope of this study [6, 20].

The refreshing of the sample results in almost total oxide removal from the PS surface and embedment of F atoms into Si layer that stimulates the ionic processes and leads to the appearance of positively charged traps, which located near the Si–SiO_2_ interface [21]. The presence of this traps leads to the electron localization and thus in *n*_cb_ reduction with *n*_tr_ rise. It resulted in the efficient self-focusing effect manifestation for the ref-PS due to the much higher polarizability of the localized carriers.

The consequent grafting of the alkyl chains C10 and C18 on the refreshed surface resulted in reduction of the self-focusing effect efficiency vs. the ref-PS for *I*<40 MW/cm^2^, being depended on the alkyl chain length. For the longer grafted chains, C18 the NLO response efficiency decreases at about 5% that is comparable with experimental error range—in comparison with an order of magnitude reduction for the PS-C10. It reflects a different level of substitution of the highly polarizable silan moieties with less efficient alkyl groups: the more effective grafting of the shorter alkyl chains on PS surface [14].

For the higher excitation level *I*>*I*_ex_ (ref-PS) ∼50 MW/cm^2^, we have observed saturation of the self-focusing effect in ref-PS in comparison with steep rise of the on-axis transmittance for the PS-C18. The last one becomes similar to the response of the chemically oxidized PS (PS-ox) probably due to the trapped carrier polarizability rise.

We should conclude that grafting of the ref-PS with C10 alkyl chains prevents both the oxidation of the PS interface and the suppression of the photoinduced absorption *Δα* and refractive index *Δn* variations (see Table 2). It is very important for the photonic and sensor applications of the functionalized PS in order to provide linear optical response regime in the wide laser excitation range.

The NLO response of the PS-ox sample can be attributed to the deep traps excitation in ultrathin highly nonstoichiometric chemically induced oxide layer. Possibility to release electrons at the Si-SiO_2_ thermally oxidized interface by light from the traps with energy levels 1.2 and 1.9 eV below the Si conduction band [22] was shown. We have observed the modification of the PS-ox NLO response from state of the trapped electron interaction with physisorbed ambient water through consequent PS-ox drying/wetting cycle at room temperature.

The dominant contribution of the trapped electron response can be explained within the comparison of the electron absorbtion cross section *σ*≤10^−16^ cm^2^ in porous silicon [23] and silicon nanocrystals [24] within the *σ*_tr_∼10^−11^ cm^2^ for the trapped ones [22]. The NLO response of the trapped electrons was resonantly enhanced due to the proximity of the laser quanta energy $\hbar \omega = 1.17$ eV to the trap depth $\hbar \omega _{\text {tr}} = 1.20$ eV [22]. An efficient NLO response of the trapped electrons for the anisotropic layers of mesoporous Si was observed in [6].

Impact of water adsorption/desorption on the NLO response can be explained in the framework of the donor-acceptor mechanism of adsorption [21]. It suggests that adsorption takes place at the defect surface sites of the undercoordinated metal atoms (the most common are dealing oxygen vacancies). Their effective charge q differs from the same of the bulk atoms. The donor molecule adsorption creates surface complex *D* ^+*δ*^ *M* ^*q*−*δ*^, where *δ* is excessive charge. Capture of the lone electron pair of H_2_O molecule by electron-acceptor center forms ≡Si:OH_2_ complex that acts as a hole trap. Trapped hole enhances the capture of the lone electron pair resulted in rise of proton-donor ability of the molecule with efficient *δ* rise due to the interband and resonant defect states optical excitation [21].

Adsorption of water induces proton conductivity in the following stages: (i) the deformed coordination-bound water molecules at the SiO_2_ surface become sources of the protons; (ii) with filling rise formation of H_2_O molecules cluster takes place around the tightly bonded molecule within proton exchange in the cluster; (iii) when merging clusters arise channels of proton conductivity. The conductivity channels can appear before formation of the multilayer water film (*θ*<1). Increase of the adsorbed layers *θ* gains in six orders of magnitude rise in protons mobility according to the relay proton transfer mechanism which is restricted by the water molecule twist [25]. Moreover, the adsorbed water molecules can diffuse through the nonstoichiometric oxide layer and consolidate at Si oxide interface [21]. Thus the photoinduced adsorption and the adsorbed molecule protonization provide essential variation of the complex surface conductivity. It corresponds to the experimental data that water adsorption on hydrophilic oxidized Si surface induces the additional bands bending at the surface, as a result, in recharge of the surface states [26, 27].

At the ambient conditions with humidity ∼75%, the absorbed water at the PS oxide shell interface can control the detuning of the trap depth position in the gap versus the $\hbar \omega $ [26, 27]. Due to the water high polarizability its surface layer produces efficient dipole at the PS-ox interface and reduces the effective detuning, which is opposite to the dielectric confinement effect [28].

Drying of the PS-ox sample at room temperature removes the physisorbed water that reduces proton mobility at the interface and release fraction of the holes trapped at donor-acceptor centers. It also provides a higher detuning of the deep trap position and the energy of laser quanta due to the highly polarizable water layer contribution decrease. Reduction of the self-focusing mechanism effects within enhancement of the delocalized holes self-defocusing contribution resulted in the refractive NLO response sign turn—the self-defocusing effect manifestation (see Fig. 6 a). The given explanation was proved by the proximity of the refractive NLO response of the dried 120-h PS-ox (dashed curve in Fig. 5 b) with the aged-PS one (curve 1 in Fig. 5 b).

Consequent water adsorption in on PS-ox surface transforms the self-defocusing phenomena (*Δn*<0) into the self-focusing (*Δn*>0) (see Fig. 6 a). Eight times enhancement of the refractive NLO response efficiency versus the PS-ox at ambient conditions due to the prolonged exposition in the saturated water vapor atmosphere was shown. From our point of view, the drastic enhancement of the proton mobility in the highly polarizable adsorbed water layer gains in the refractive NLO efficiency enhancement within delocalized holes concentration reduction due to the trapping at the donor-acceptor centers.

We have observed indirect confirmation of the signature of the polymolecular adsorption in pores that can transform in capillary condensation [29], within significant reduction of elastic light scattering efficiency (see Table 3) due to the optical density contrast decrease at the developed oxide-water interface.

The obtained results of water adsorption/desorption on the PS-ox surface are in a good agreement with the ones observed recently [3, 26, 27] and indicate that novel type transducer/sensor can be proposed on the base of the refractive NLO response efficiency monitoring within the self-action of picosecond laser pulses at 1064 nm.

## Conclusions

The nonlinear optical response due to the self-action of the picosecond range pulsed laser radiation at 1064 nm in the functionalized porous silicon was studied. It was shown that the PS surface functionalization—refreshing/oxidation/alkylation—manifests in laser beam self-focusing (*Δn*>0) effect versus the self-defocusing one (*Δn*<0) observed in the aged PS. The NLO response magnitude was sensitive to the alkyl chain length.

We have observed the dominant contribution of the refractive NLO response over the absorptive one |Re(*χ* ^(3)^)/Im(*χ* ^(3)^)|∼10^3^−10^4^ that is promising for the photonics applications. We have observed nonlinear Fabry-Perot interferometer effect manifestation in the photoinduced total transmittance dependencies in the initial laser excitation range due to the efficient NLO refractive index variation.

It was shown that grafting of the ref-PS with C10 alkyl chains prevents both the oxidation of the PS interface and the suppression of the photoinduced absorption *Δα* and refractive index *Δn* variations. The last is very important for the PS applications in photonics and sensor fields in order to provide linear response regime in the wide laser excitation range of the PS.

The impact of the water adsorption on NLO properties of chemically oxidized porous silicon was studied. It was shown that under the initial conditions (air humidity ∼75%), the sample demonstrate the self-focusing effect with Re(*χ* ^(3)^)∼4.1×10^−9^ esu. The water desorption from the sample’s surface causes the refractive NLO response sign turn to self-defocusing. For the dried sample, the Re(*χ* ^(3)^) magnitude was about −4.7×10^−9^ esu. The consequent exposition of the dried sample in the saturated water vapors at room temperature induces the reverse sign to turn to self-focusing and the about 8 times enhancement of the refractive NLO response efficiency Re(*χ* ^(3)^)∼5×10^−8^ esu. The obtained results were explained in terms of the resonant excitation of deep defects with the laser pulses in the ultrathin highly nonstoichiometric chemically induced oxide layer at the PS developed interface.

On the basis of the obtained results, we suggest to utilize the picosecond range pulsed laser radiation self-action effects monitoring at 1064 nm for the PS surface diagnostics and sensing applications.
